# Why Does Kazakhstan Need New Scientific Journals?

**DOI:** 10.5195/cajgh.2014.141

**Published:** 2014-07-10

**Authors:** Shalkar Adambekov, Galiya Dosmukhambetova, Gani Nygymetov, Ronald LaPorte, Faina Linkov

**Affiliations:** 1 Center for Life Sciences, Nazarbayev University, Astana, Kazakhstan; 2 Center for International Programs, Astana, Kazakhstan; 3 Department of Epidemiology, University of Pittsburgh, Pittsburgh, PA; 4 Magee-Womens Research Institute, University of Pittsburgh, Pittsburgh, PA

Kazakhstan is young country located in the heart of the Eurasian continent. It was an integral part of the Soviet Union until 1991 and was primarily known for oil, mining, agriculture, as well as nuclear bomb testing. During the Soviet Era, Kazakhstan underwent industrialization and the country’s economy shifted from purely agricultural to partially industrial. To provide cadre for the new industries, a large number of educational and scientific institutions were established; however, most of the scientific research was concentrated in the European part of the Soviet Union and was heavily government controlled.^1^ Scientific developments were Moscow-oriented. Kazakhstani scientists published almost exclusively in Russian language journals and periodicals. As a result of this scientific isolation, Kazakhstani scientists were not known outside of the former USSR region, which became evident after Kazakhstan gained its independence. Currently, Kazakhstan is seeking integration into the international scientific arena, which is one of the goals of the 2050 development strategy.

As part of this strategy, Kazakhstan is aiming to develop a knowledge and innovation-based economy in addition to harnessing its rich mineral and oil resources. Governmental attention is focused on the modernization of Kazakhstani research, pushing scientific innovation and producing competition by establishing new universities, schools, and research centers. In addition, a governmental scholarship program is established in Kazakhstan, which is responsible for training young specialists in the world’s leading universities. The financing of the science sector is constantly increasing, albeit the portion of GDP spent on R&D is less than 0.25%, compared with an average 1.7% in developed countries.^2^ The transition from a resource-based economy to a science-oriented state is slow, and requires passing many important milestones. One of the most important benchmarks in establishing a knowledge based economy is achieving competitive scientific productivity. The currency of scientific productivity is articles published in internationally recognized, English-language journals, which are available to a wide readership.

In the past 25 years, Kazakhstan transitioned from being part of an industrialized country in the Soviet era to a developing country in 1990s, and is now a developed country in the 2010s. This creates an unique public health environment with an ongoing epidemiologic transition,^3^ coupled with the double burden of chronic and infectious diseases. Excess morbidity and mortality associated with tuberculosis^4^ and increasing life expectancy take place in parallel. It makes Kazakhstan an interesting model for public health and medical research.

There are a number of prominent medical research centers in Almaty, Astana, and Karaganda that are equipped to address these problems; we can hypothesize that Kazakhstan can potentially produce interesting and relevant research. However, according to the bibliographic database Scopus, only around 7% of articles published by Kazakhstani scientists were in the areas of medicine and public health. It does not mean that Kazakhstani specialists produce less research in this area, but they evidently publish less in international journals. The exact reasons for this discrepancy are not known, but one might hypothesize that major reasons include a lack of English language skills, training in research methodology, and the aforementioned scientific isolation, which is especially relevant for conservative medical workers. This trend is not unique for Kazakhstan, but for all of Central Asian countries. Whereas most post-Soviet countries are spread between 50^th^ and 100^th^ place in the list of countries according to the number published documents, Central Asian countries such as Uzbekistan, Kazakhstan, Kyrgyzstan, Tajikistan and Turkmenistan are in 82^nd^, 94^th^, 143^rd^, 148^th^, and 183^rd^ places, respectively. Despite a large number of general and specialized journals Kazakhstan and Central Asia represent a blank spot on the scientific publishing map.

The situation with English language and research methodology can be solved by modern education, workshops, and training courses. However the problem of scientific isolation is not that easily fixed. In the absence of recognized authors and institutions, the question of research credibility is crucially important. It is usually difficult to publish research if one does not have an established name supporting one’s manuscript, which is a problem for Kazakhstan authors.

To solve this situation, two major approaches can be applied. The first is to establish international collaboration and hire foreign professors; such is the case, for example, of Nazarbayev University – a newly established Kazakhstani University based on the western standards of education and research. The second approach is to train the next generation of researchers in countries that have high publication rates, which is the primary mission of the “Bolashak” scholarship. The third approach is to establish a platform for publishing research from Kazakhstan and Central Asia, such as the establishment of a new scientific journal. If one examines the statistics, countries such as the US and the UK own the top 20 highest H Index ranking journals. Overall, among 20,544 journals referenced in Scopus, 5,605 are of US origin and 5,036 are published in UK. Thus, 50% of all scientific documents indexed in Scopus are published in these countries. To be successful, the new journal must follow a simple rule- be an English language, peer-reviewed journal, but be primarily focused on the Central Asian research community to avoid the “credibility” problem. The Central Asian Journal of Global Health ( cajgh.pitt.edu ) has been developed in collaboration between experts at Nazarbayev University and the University of Pittsburgh with the goal of ending scientific isolation and allowing Kazakhstan scientists to publish their research discoveries in an English language medium.

In conclusion, Kazakhstan is a progressively developing country aimed to become a regional power through the establishment of a diversified economy based on knowledge and innovation. However, to achieve this, Kazakhstan needs to overcome several barriers, one of which is the scientific isolation that resulted from being part of the Russian oriented science system. Establishing English language peer-reviewed journals is a one way of tackling this problem and could help emerging Kazakhstani scientists make significant contributions to the international scientific arena.

## Figures and Tables

**Figure 1:**
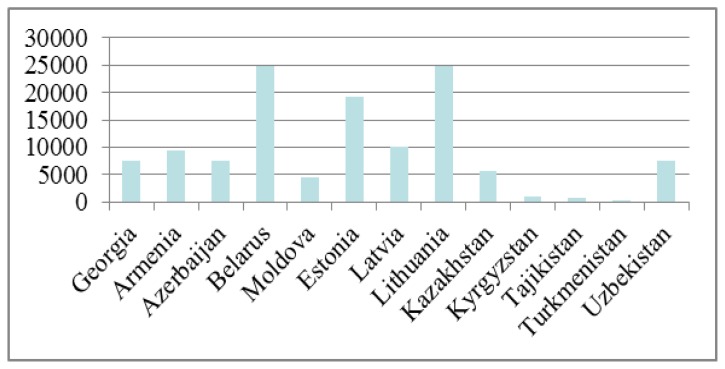
The total number of publications for 13 former Soviet Union countries for 1996–2012
